# COVID-19-Related Publications by Hospitalists in the United States

**DOI:** 10.7759/cureus.35553

**Published:** 2023-02-27

**Authors:** Nicole Bonk, Richard Elias, Andrea White, Shandra Payne, Casey Wagner, Farah Kaiksow, Ann Sheehy, Andrew Auerbach, Valerie M Vaughn

**Affiliations:** 1 Hospital Medicine, University of Wisconsin, Madison, USA; 2 Hospital Medicine, Mayo Clinic, Rochester, USA; 3 Hospital Medicine, University of Utah, Salt Lake City, USA; 4 Hospital Medicine, Utah Valley University, Orem, USA; 5 Internal Medicine, University of California San Francisco, San Francisco, USA; 6 Internal Medicine, Division of General Internal Medicine, University of Utah, Salt Lake City, USA

**Keywords:** promotion, authorship, covid-19 publications, academic research, hospital medicine

## Abstract

Objective

To determine the degree to which hospitalists published academic manuscripts related to COVID-19 during the first year of the pandemic.

Patients and methods

The study was a cross-sectional analysis of the author's specialty, defined by byline or professional online biography, from articles related to COVID-19 published between March 1, 2020, and February 28, 2021. It included the top four internal medicine journals by impact factor:* New England Journal of Medicine, Journal of the American Medical Association, Journal of the American Medical Association Internal Medicine*, and* Annals of Internal Medicine*. Participants were all United States (US)-based physician authors contributing to COVID-19 publications. Our primary outcome was the percentage of US-based physician authors of COVID-19 articles who were hospitalists. Subgroup analyses characterized author specialty by authorship position (first, middle, last) and article type (research vs. non-research).

Results

Between March 1, 2020, and February 28, 2021, the top four US-based medical journals published 870 articles related to COVID-19 of which 712 articles with 1940 US-based physician authors were included. Hospitalists accounted for 4.2% (82) of authorship positions including 4.7% (49/1038) of authorship positions in research articles and 3.7% (33/902) of authorship positions in non-research articles. First, middle, and last authorship positions were held by hospitalists at 3.7% (18/485), 4.4% (45/1034), and 4.5% (19/421) of the time, respectively.

Conclusions

Despite caring for a large number of patients with COVID-19, hospitalists were rarely involved in disseminating COVID-19 knowledge. Limited authorship by hospitalists could constrain the dissemination of inpatient medicine knowledge, impact patient outcomes, and affect the academic promotion of early-career hospitalists.

## Introduction

During the first year of the COVID-19 pandemic, from March 2020 through February 2021, there were 154,731 COVID-19-related hospitalizations in the United States (US) [1]. With this crisis, came an explosion of published work as healthcare providers tried to share what they had learned from researching and caring for patients with this novel disease: a search of the MeSH (medical subject headings) term “COVID-19” in PubMed.gov reveals 93,199 articles in 2020 and 136,496 articles in 2021.

Hospitalists, or physicians who provide dedicated inpatient care, provided the majority of inpatient COVID-19-related care in the US during the pandemic [2]. The subspecialty of hospital medicine has grown quickly over the last few decades. From 1996 through 2016, the number of hospitalists in the US grew from several hundred hospitalists to over 50,000 [3]. Hospitalists now outnumber the second biggest subspecialty in internal medicine, cardiology, with 22,000 physicians. With this rapid growth has come challenges. Compared to other specialties, hospitalists are primarily clinical, often have large turnover with shorter careers, and are less likely to publish or be promoted [4].

For many hospitalists, the COVID-19 pandemic has meant working longer hours, seeing more patients, placing themselves at higher personal risk, and experiencing more burnout [5]. In addition, because of their expertise in hospital operations, many hospitalists were called upon to lead surge responses [6]. These positions at the forefront of inpatient care likely enabled hospitalists to gather great clinical insight into managing acute COVID-19. Ideally, this insight would have translated to the dissemination of knowledge through peer-reviewed publications. However, there are pre-existing disparities in research infrastructure between hospital medicine and other subspecialties [7]. It is unclear if hospitalists shared their first-hand expertise and knowledge through peer-reviewed publications. Thus, we aimed to examine the author's specialty, with a focus on hospital medicine, in publications related to COVID-19 in US-based high-impact internal medicine journals.

A version of this information was previously presented as a poster at the Society of Hospital Medicine 2022 Converge Conference in Nashville, Tennessee on April 8, 2022.

## Materials and methods

Data source

To identify published articles on COVID-19, we conducted a manual review of the tables of contents of the top four highest-impact US-based internal medicine journals *New England Journal of Medicine (NEJM)*, *Journal of the American Medical Association (JAMA)*, *Journal of the American Medical Association Internal Medicine (JAMA-IM)*, and *Annals of Internal Medicine (Annals)*. These four journals were chosen as the most representative of high-quality, internal medicine journals so as to avoid skewing the data toward any medical specialty.

**Figure 1:**
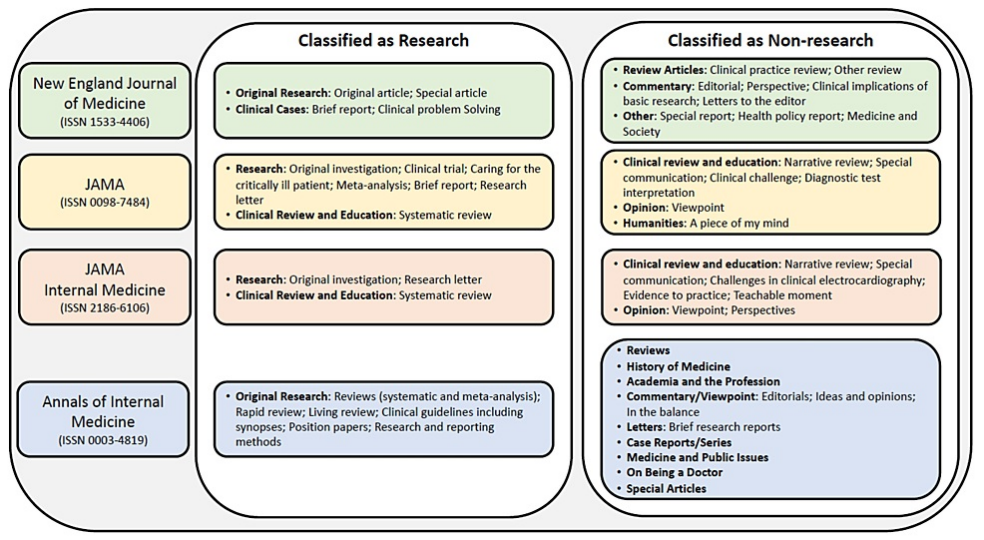
Classification of articles as research versus non-research JAMA: Journal of the American Medical Association

Two researchers independently reviewed the tables of contents of each journal issue, published between March 1, 2020, through February 28, 2021, to identify eligible articles. Articles were eligible for inclusion if they had specific keywords in the title related to COVID-19 (i.e., COVID-19, SARS-CoV-2, Coronavirus Disease 2019, 2019-nCoV, pandemic). Because of challenges in determining subspecialties internationally, only articles that had a US-based first or last author were included. Articles were categorized as research (original investigation or systemic review) or non-research (see Figure 1).

Some article types were excluded such as opinion pieces, graphic and audio publications, and replies to the editor. Discrepancies in eligibility criteria were resolved through discussion with a third reviewer serving to break the tie.

Data collection and definitions

For each included article, we recorded authorship position (e.g., first, middle, last) and, for US-based physician authors, we identified physician specialty. The specialty was based on author byline (if noted), otherwise online biographies were reviewed for specialty, division, or fellowship. Data entry occurred independently in duplicate using a REDCap survey, with discrepancies resolved by consensus.

Historical comparison

To assess whether COVID-19 publication rates by hospitalists were similar to pre-pandemic rates of publication by hospitalists, we also collected authorship data on articles related to pneumonia or sepsis that were published in the same four journals in the immediate pre-pandemic era (March 1, 2019, through February 28, 2020). Pneumonia and sepsis, similar to COVID-19, are both diseases where hospitalists - but also other specialists (e.g., pulmonary critical care, emergency medicine, and infectious diseases) - serve as frontline clinicians for inpatient care. For this comparison, articles were identified if they had the keyword “pneumonia” or “sepsis” in the title and otherwise met inclusion criteria (e.g., US-based authorship criteria).

Primary outcome

Our primary outcome was the percentage of US-based physician authors of COVID-19 articles who were hospitalists. Hospitalists were defined as any mention of “hospital medicine” or “inpatient medicine” as a specialty. Family medicine, pediatric, and internal medicine physicians were included if they were publicly identified as hospitalists. If a physician was listed as a primary care physician (e.g., “family medicine,” “general internal medicine”), but not listed in any online biography as an inpatient physician or hospitalist, they were presumed to be non-hospitalist. If a physician was listed as being both a hospitalist and another specialty, they were included as a hospitalist.

To allow comparison across specialties, non-hospitalist physicians were classified as non-hospitalist primary care (i.e., general internal medicine, family medicine, and pediatrics), internal medicine subspecialty (i.e., infectious disease, pulmonary critical care, gastroenterology, rheumatology, endocrinology, hematology and oncology, cardiology, geriatrics, allergy and immunology, nephrology, or hospice/palliative care), or non-internal medicine specialty (i.e., anesthesia, emergency medicine, surgery, neurology, dermatology, obstetrics and gynecology, pathology, radiology, medical genetics, ophthalmology, physical medicine, and rehabilitation, psychiatry, or pain specialty).

Subgroup analyses

Given the importance of first and last authorship positions in promotion, we examined the percentage of US-based physician authors of COVID-19 articles who were hospitalists by authorship position divided into first, middle, and last authorship positions.

Given the importance of research articles compared to other article types for academic promotion, we also examined the percentage of US-based physician authors of COVID-19 articles who were hospitalists after dividing article types into two categories: (a) research (e.g., original investigation, systematic review) and (b) non-research (e.g., editorial, viewpoint). A detailed breakdown of article types by journal can be found in the Appendix.

Data analysis

We characterized physician specialty across all included COVID-19 articles using descriptive statistics. Using chi-squared or fisher-exact tests, as appropriate, we compared the percentage of US-based physician authors of COVID-19 articles who were hospitalists vs. non-hospitalists. Similar methods were used to characterize the author's specialty in subgroup analyses. Finally, we used fisher-exact tests to compare the percentage of US-based physician authors of COVID-19-related publications who were hospitalists to authors of sepsis or pneumonia articles in the year prior to the pandemic. A P-value <0.05 is considered significant. We followed Enhancing the QUAlity and Transparency Of health Research (EQUATOR) reporting guidelines.

Regulatory requirements

This study used only publicly available data and received nonregulated status from the University of Wisconsin Institutional Review Board.

## Results

Article types

**Figure 2:**
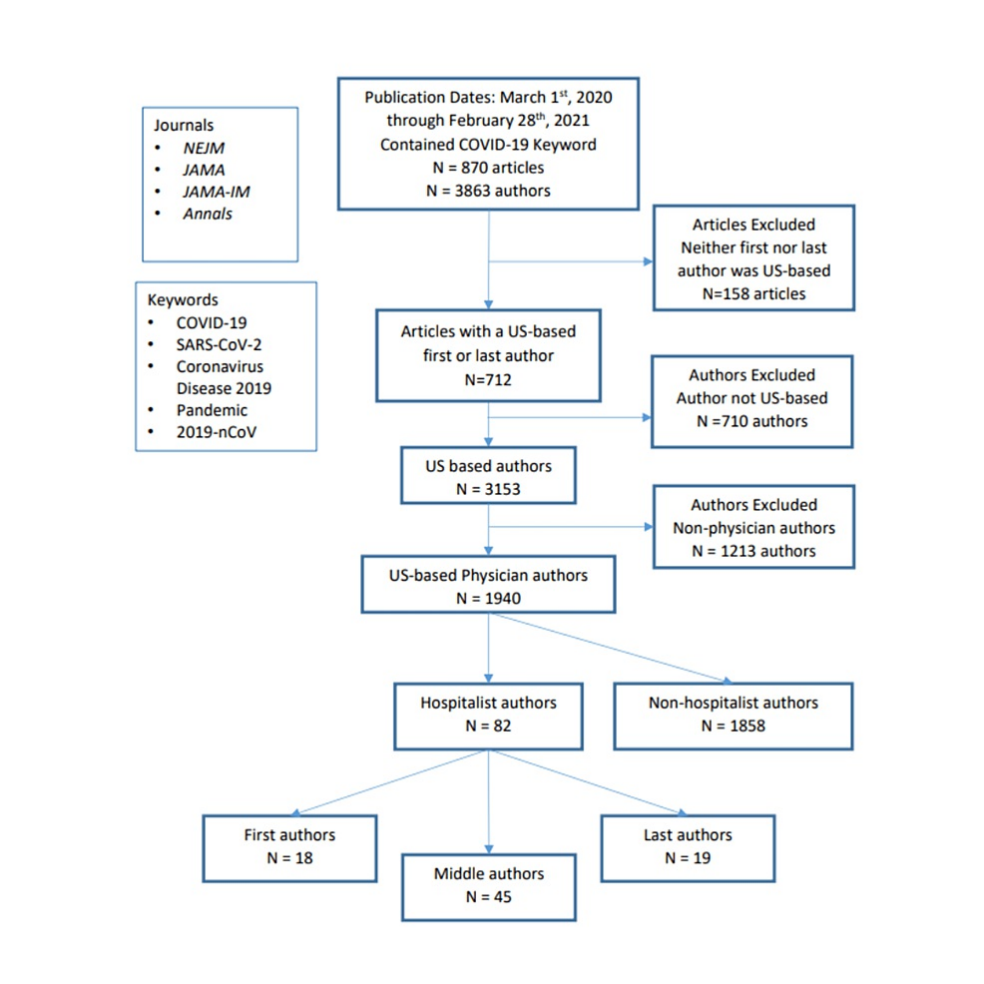
Study flow diagram NEJM - New England Journal of Medicine; JAMA - Journal of the American Medical Association; JAMA-IM - Journal of the American Medical Association Internal Medicine; Annals - Annals of Internal Medicine

Between March 1, 2020, and February 28, 2021, the top four US-based medical journals published 870 articles with a COVID-19-related keyword. Of those, 158 did not have a US-based first or last author and were excluded. Therefore, 712 articles met the final inclusion criteria (see Figure 2 for the inclusion flowchart). Of these, 170 (23.9%) were original research articles and 542 (76.1%) were non-research articles (i.e., clinical review, education, commentary, or editorial). Of the 170 research articles, 20.0%, 38.2%, 17.6%, and 24.1% were from NEJM, JAMA, JAMA IM, and Annals, respectively. For non-research articles, 26.6%, 52.7%, 6.5%, and 14.2% were from NEJM, JAMA, JAMA IM, and Annals, respectively.

Authorship analysis

Across 712 included articles, there were 3,153 US-based authors. Of these, 61.5% (1,940) were physicians and 38.5% (1,213) were non-physician authors. In total, there were only 82 hospitalist authors, accounting for 2.6% (82/3,153) of all US-based authors and 4.2% (82/1,940) of all US-based physician authors. Thus, US-based physician authors were far more likely to be non-hospitalists than hospitalists (P<0.001; see Table 1 for details). For reference, US-based physician authors of COVID-19 manuscripts were similarly likely to be cardiologists (5.5% (107/1,940), P=0.06) - a specialty that did not typically provide frontline care - and far more likely to be pulmonary/critical care physicians (9.0% (174/1,940), P<0.001) - a smaller specialty that commonly cared for hospitalized patients with COVID-19. Across journals, the percentage of US-based authors who were hospitalists was 3.1% (21/677) in NEJM; 3.5% (24/686) in JAMA; 4.3% (10/232) in JAMA-IM; 7.8% (27/345) in Annals.

**Table 1:** Total authors of COVID-19 publications from 3/1/20 through 2/28/21 * Hospitalists were identified as internal medicine, pediatric, or family medicine physicians who identified themselves as hospitalists for at least part of their practice. Non-hospitalist physicians were identified as any MD, DO, MBBS, MBChB, or MBBCh who did not list hospitalists as part of their practice.

n=all authors	Non-physician authors	Physician (all) authors	Hospitalists^*^	Non-hospitalist physicians	% of physician authors who are hospitalists
Original research, n=1870	832	1038	49	989	4.72%
First author, n=165	56	109	5	104	4.59%
Middle author, n=1538	732	806	38	768	4.71%
Last author, n=167	44	123	6	117	4.88%
Non-research, n=1283	381	902	33	869	3.66%
First author, n=538	162	376	13	363	3.46%
Middle author, n=356	128	228	7	221	3.07%
Last author, n=389	91	298	13	285	4.36%
All article types, n=3153	1213	1940	82	1858	4.23%

**Figure 3:**
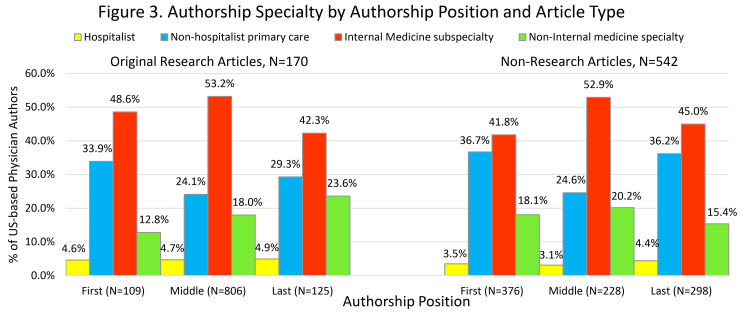
Authorship specialty by authorship position and article type Non-research articles include clinical reviews, education, commentary, and editorial articles.

Of the 82 hospitalist authors, 22% (18) were first authors, 55% (45) were middle, and 23% (19) were last authors. Hospitalists represented 4.7% (49/1,038) of US-based physician authors of original research and 3.7% (33/902) of non-research publications. For reference, cardiologists represented 6.1% (63/1,038) of US-based authors on original research and 4.9% (44/902) of non-research publications. Across all article types and authorship positions, US-based physician authors were more likely to be non-hospitalists than hospitalists (P<0.001 for all comparisons; see Figure 3 for details).

In the year prior to the COVID-19 pandemic, there were 15 articles published on sepsis and 11 articles published on pneumonia. After exclusions, there were 102 US-based physician authors, of whom 6.9% (7/102) were hospitalists. Of first, middle, and last US-based physician authors, 12.5% (2/16), 1.4% (4/70), and 6.4% (1/16) were hospitalists, respectively. Though numerically higher, the proportion of US-based first or last authors of pneumonia or sepsis articles and who were hospitalists (6.9% (7/102)) was not statistically different than for COVID-19 articles (4.1% (37/906), P=0.152).

## Discussion

Of the 1,940 US-based physician authors that published articles related to COVID-19 between March 1, 2020, and February 28, 2021, in the top four internal medicine journals, only 4.2% were hospitalists. This is despite the fact that hospitalists were the primary inpatient providers and clinician leaders during the COVID-19 pandemic: unpublished data from the Mi-COVID19 collaborative quality initiative demonstrated that of 2784 patients hospitalized with COVID-19 across 35 Michigan Hospitals, 1592 (57.2%) had a hospitalist as the main provider at some point [2]. The lack of publication by hospitalists may have limited critical dissemination of COVID-19-related knowledge during the pandemic. Furthermore, limited academic dissemination by hospitalists could worsen disparities in the promotion of hospital medicine faculty with a particular impact on early career hospitalists who may have volunteered to provide frontline care during the COVID-19 pandemic.

Dissemination of knowledge

Hospital medicine is the largest internal medicine specialty and one of the largest groups of frontline clinicians for patients hospitalized with COVID-19, yet only 4.2% of included authors in this study were identified as hospitalists. For comparison, cardiologists (who have approximately half the number of practicing physicians as hospitalists and generally were not primary providers for patients with COVID-19) [4] represented 5.5% of US-based physician authors, and pulmonary/critical care physicians (who were also common inpatient providers for patients with COVID-19 but are a much smaller specialty) represented 9.0% of US-based physician authors. It is concerning that hospitalists providing frontline care and gaining initial clinical experience with COVID-19 were not positioned to establish the research trials that disseminated knowledge quickly and effectively. Such impaired dissemination could result in limited knowledge sharing from hospitals initially hit with COVID-19 to others who experienced later waves. Similarly, the predominance of specialty-focused publications could have shifted the focus from more holistic practices for patient safety that we know help hospitalized patients who are critically ill. For example, 34.8% of patients missed two or more days of venous thromboembolism prophylaxis early in the pandemic [6], and healthcare-associated infections saw a dramatic increase, with as many as three to four times increased rate of central line-associated bacterial infection [8]. Hospitalists helping to disseminate this awareness and knowledge early on may have helped protect patients from well-known preventable complications.

Academic advancement

In addition to its importance for scientific advancement, the dissemination of knowledge and the development of a national reputation is typically required for academic advancement. The primary “currency” of academic advancement is peer-reviewed publications with a particular emphasis on research publications and first or last authorship positions. In a 2021 study of the top 25 internal medicine residency programs, 51.4% of hospitalist faculty had zero publications and only 11.7% of academic hospitalists had been promoted to associate or full professor [9]. In comparison, a 2014 study of academic cardiologists reported that 28.2% were full professors [10]. Unfortunately for early career hospitalists, the lack of publications - coupled with increased clinical duties that are not normally considered in promotion - may have served to increase disparities in the academic promotion of hospital medicine faculty. Though some have argued for alternative metrics and redesigning curriculum vitae to acknowledge COVID-19-related duties [11], to our knowledge, no institution has yet adjusted its promotion criteria to incorporate atypical COVID-19 duties.

Limitations of our study

Our study has limitations. First, as hospital medicine is primarily a US-based specialty, our findings do not apply outside the US. For example, in 2017 the Society of Hospital Medicine had 15,000 US-based members and only 126 international members [12]. Second, there may be other journals (e.g., *Journal of Hospital Medicine*, *Journal of General Internal Medicine*) in which hospitalist physicians are more likely to publish or that may be more interested in the type of scholarship (e.g., quality improvement, health services research) that hospitalists produce. We limited the study to the top four internal medicine journals by impact factor and intentionally chose not to include specialty journals to avoid skewing the data towards one specific specialty. Third, we may have under-identified hospitalists as not all self-identify as hospitalists and may be housed in other divisions (e.g., general internal medicine). Similarly, as our data were gathered from authors’ online biographies, we may have misclassified specialties. Fourth, a true pre/post analysis was impossible because there were no COVID-19 articles prior to the pandemic. We elected to compare pneumonia and sepsis publications as the closest available approximation to COVID-19, however, there are likely publication differences attributable to the differences between diseases. Furthermore, our study is underpowered to truly compare pre- vs. post-pandemic publications given the limited number of sepsis and pneumonia publications identified in the immediate pre-COVID period. Strengths include a review in duplicate, a search in multiple places for specialty, and the inclusion of a historical control group.

Implications

Hospitals must invest in research infrastructure to encourage growth in hospital medicine research. Despite the explosive growth of clinical hospitalists over the past 25 years, the research infrastructure for hospital medicine has not kept pace. Thus, hospitalists were not positioned to disseminate knowledge or participate in clinical research related to the care being provided. Hospitalists are ideally positioned to provide critical clinical insight and perform other patient-facing research duties, such as recruitment for clinical trials. As many hospitalist programs are still primarily composed of early and mid-career faculty, and there is an imperative for clinical production, the specialty may need to employ alternative infrastructure models to support hospitalists’ academic development. For example, the University of Washington hired an academic coach to support hospitalist academic production and the 0.50 FTE position contributed to producing 63 publications [13]. Medical schools should consider allowing COVID-19-related non-research duties, such as additional clinical care and administrative roles, to count toward academic promotion. During the pandemic, many hospitalists worked extra clinical effort to care for the rising hospital census - including service in administrative roles or even as intensivists. Unless this extra work is considered in promotions, hospitalists risk being further delayed compared to non-frontline clinicians who may have had more time and thus opportunity for research during the pandemic.

## Conclusions

Despite being primary frontline providers of inpatient medical care for patients hospitalized with COVID-19 - and one of the largest medical specialties - hospitalists were rarely involved in disseminating COVID-19 knowledge. Limited authorship by hospitalists could constrain the dissemination of inpatient medicine knowledge, impact patient outcomes, and affect the academic promotion of early-career hospitalists. Improved research infrastructures within hospital medicine programs may help avoid this problem in the future.
